# Standardizing the measurement of parasite clearance in falciparum malaria: the parasite clearance estimator

**DOI:** 10.1186/1475-2875-10-339

**Published:** 2011-11-10

**Authors:** Jennifer A Flegg, Philippe J Guerin, Nicholas J White, Kasia Stepniewska

**Affiliations:** 1 WorldWide Anti-malarial Resistance Network (WWARN) and Centre for Tropical Medicine, Nuffield Department of Clinical Medicine, University of Oxford, Churchill Hospital, Old Road, Oxford, OX3 7LJ, UK; 2 Mahidol-Oxford Tropical Medicine Research Unit, Faculty of Tropical Medicine, Mahidol University, Bangkok, Thailand; 3 Centre for Tropical Medicine, Nuffield Department of Clinical Medicine, University of Oxford, Churchill Hospital, Old Road, Oxford, OX3 7LJ, UK

**Keywords:** malaria, regression analysis, parasite clearance, artemisinin resistance, drug resistance

## Abstract

**Background:**

A significant reduction in parasite clearance rates following artesunate treatment of falciparum malaria, and increased failure rates following artemisinin combination treatments (ACT), signaled emergent artemisinin resistance in Western Cambodia. Accurate measurement of parasite clearance is therefore essential to assess the spread of artemisinin resistance in *Plasmodium falciparum*. The slope of the log-parasitaemia *versus *time relationship is considered to be the most robust measure of anti-malarial effect. However, an initial lag phase of numerical instability often precedes a steady exponential decline in the parasite count after the start of anti-malarial treatment. This lag complicates the clearance estimation, introduces observer subjectivity, and may influence the accuracy and consistency of reported results.

**Methods:**

To address this problem, a new approach to modelling clearance of malaria parasites from parasitaemia-time profiles has been explored and validated. The methodology detects when a lag phase is present, selects the most appropriate model (linear, quadratic or cubic) to fit log-transformed parasite data, and calculates estimates of parasite clearance adjusted for this lag phase. Departing from previous approaches, parasite counts below the level of detection are accounted for and not excluded from the calculation.

**Results:**

Data from large clinical studies with frequent parasite counts were examined. The effect of a lag phase on parasite clearance rate estimates is discussed, using individual patient data examples. As part of the World Wide Antimalarial Resistance Network's (WWARN) efforts to make innovative approaches available to the malaria community, an automated informatics tool: the parasite clearance estimator has been developed.

**Conclusions:**

The parasite clearance estimator provides a consistent, reliable and accurate method to estimate the lag phase and malaria parasite clearance rate. It could be used to detect early signs of emerging resistance to artemisinin derivatives and other compounds which affect ring-stage clearance.

## Background

Anti-malarial drug resistance is a major cause of preventable mortality [1] and poses the main threat to current global efforts to control and eliminate malaria [2]. Replacement of failing mono-therapy with highly effective artemisinin combination therapy (ACT) and increased delivery of effective vector control measures have reversed the increasing malaria mortality trend observed in the 1980s and 1990s. ACT has now become the recommended first line treatment for falciparum malaria in nearly all malaria affected countries [3].

The recent emergence of artemisinin resistance in *Plasmodium falciparum *malaria in Western Cambodia represents a considerable threat to global health [4-6]. A significant reduction in the rates of parasite clearance following treatment with artesunate and increased failure rates following artemisinin combination treatments (ACT) provided definitive evidence of resistance in that region [5,7,8]. There is increasing concern that artemisinin resistance has spread westwards [9]. No molecular marker of artemisinin resistance has yet been identified, and *in vitro *assessments have given contradictory results [5-7]. The clinical phenotype of slow parasite clearance remains to date the only way to define artemisinin resistance reliably [10].

The unique ability of the artemisinin derivatives to accelerate the clearance of ring stage infected erythrocytes is their pharmaco-dynamic hallmark [11,12]. Accurate measurement of the rate of parasite clearance is necessary to assess artemisinin susceptibility *in vivo*. Most *in vivo *assessments measure parasitaemia either daily, or only on days D0, D2 and D3, as recommended in the current World Health Organization (WHO) guidelines [6]. In most studies, the exact timing of the parasite count is not recorded and could vary by several hours depending on the timing of the visit to the clinic at inclusion and during follow-up visits. The proportion of patients with persistent parasitaemia at D3 after ACT provides a useful indicator which can be used as a simple and readily obtained measure to exclude resistance, but not to define resistance [13]. It is too inaccurate for precise assessment as it is dependent on pre-treatment parasite density, precise timing of the sample, and it requires large sample sizes for precise estimates. In previous studies, with more frequent sampling, several different methods of analysing and measuring parasite clearance have been employed but systematic analytical approaches to measurement have not been taken.

**Figure 1:**
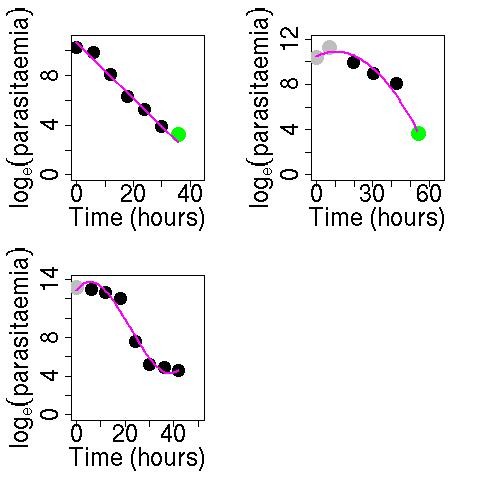
**Typical linear, quadratic or cubic-shaped log(parasitaemia)-time profiles (polynomial fit shown in pink line) in falciparum malaria**. Black points represent the data, green points represent censored values and red points represent points removed in the cleaning process.

The slope of the log-parasitaemia *versus *time relationship is considered to be the most robust measure of anti-malarial effect, but there are several potential sources of error if a straight line is fitted to all log- transformed parasite measurements, irrespective of the shape of the relationship with time [14]. Changes in parasitaemia, particularly during the first few hours after anti-malarial drug administration, are affected by the age distribution of the parasite population [15,16]. If the majority of parasites in the peripheral blood film are very young then it is likely that schizogony is still taking place at the time of drug administration. Newly parasitized erythrocytes will appear in the circulation shortly afterwards. This may be reflected by a stable density or even an increase in peripheral parasitaemia, despite effective treatment. In these cases, the fitted line to the log-parasitaemia *versus *time data will underestimate the parasite counts at the initial time period and as a result the slope of this line will underestimate the parasite clearance. It is more appropriate to fit either a quadratic model in the case where a lag phase is followed by a rapid decline in parasite number or a cubic model in the case where a rapid decrease in parasitaemia is followed by a flattening off of the parasitaemia-time profile. Figure 1 shows typical linear, quadratic and cubic-shaped log (parasitaemia)-time profiles (polynomial fits are shown in the pink line).

In an attempt to standardize parasite clearance assessments from parasitaemia-time profiles in *P. falciparum *malaria, and facilitate epidemiological investigations of artemisinin resistance a method of identifying the initial lag phase and calculating a slope of log-parasitaemia changes over time after this lag phase has been developed and implemented into the parasite clearance estimator (PCE). The algorithm chooses between three models (log-linear, log-quadratic and log-cubic) and also provides automation of the "cleaning" of parasite count data before model fitting. The PCE may be used for any anti-malarial drug assessment, although it has been tested in data from patients treated with artemisinin derivatives and various ACT.

In this paper, the details of a simple calculator (PCE) that provides measures of parasite clearance from serial parasite count data are presented including the background, terminology, method of use, and examples of its application to real data.

## Methods

Parasite clearance following any effective anti-malarial treatment is a first order process, resulting in killing of a fixed fraction of the parasite population in each asexual cycle, and can be considered as the reciprocal of parasite multiplication [11,17]. The predominant relationship between log-transformed parasite density and time is generally linear [14,18,19]. Key terminology in relation to parasite clearance estimation is presented below.

**Detection limit - **for low parasite densities the thick blood smear is used and the number of parasites (asexual forms only i.e. trophozoites) are counted against the number of white blood cells (usually 200 or 500). The detection limit obviously depends on the number of white blood cells counted. To estimate parasitaemia per microlitre the following formula is used:

$$\begin{array}{c} \text{If }\omega \text{ is the number of white cells counted then}\\ \text{Parasitaemia per microlitre }=\text{ number of parasites per slide }\times \text{ white blood cell count }∕\;\omega \end{array}$$

Ideally the white blood cell (wbc) count is measured by an automated cell counter or manually. If this is not available then the counts are assumed to be 8,000/uL.

When the white blood cell count is assumed 8,000 for all patients, then the detection limit will be 40/uL for counting per 200 wbc and 16/uL for counting per 500 wbc.

**Negative parasite slide **- when no parasites are seen while the full number of white cells have been counted then the parasite count is recorded as zero. Of course this means only that the count is below the limit of detection, although it is often reported or modeled as 0/uL.

**Outliers - **parasite counts which are not biologically possible or are highly unlikely based on other parasite measurements in the same individual. These often result from transcription errors.

**Lag phase - **initial part of the parasite clearance profile which has a much flatter slope that the remaining part of the profile. It is important to note that a lag phase is not observed in all profiles.

**Tail - **terminal part of the parasite clearance profile when parasitaemia remains close to the detection limit (i.e. a few parasites per slide) and does not decrease over a number of measurement time-points. Tails are not observed in all profiles.

**Figure 2:**
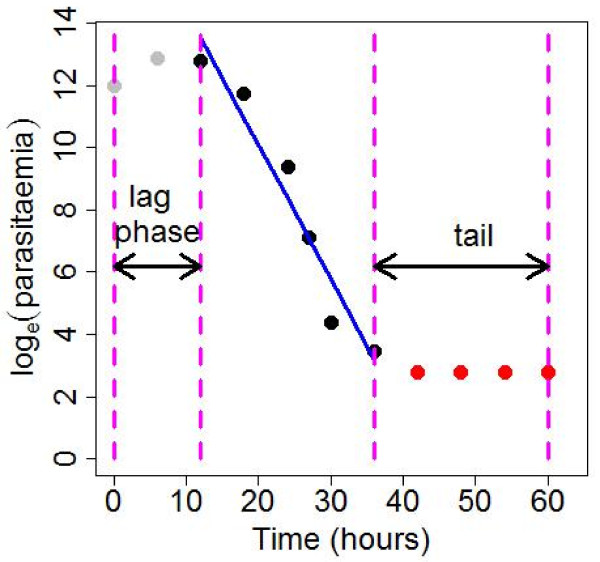
**The effect of lag phase and tail exclusion on the calculation of the clearance rate constant**.

**Clearance rate constant - **the main part of parasitaemia clearance follows a first order process and therefore the fraction by which parasite count falls per unit time is constant. If parasitaemia at time t is given by P_t _= P_0_exp(-K×t), where P_0 _is the initial parasitaemia, then the fractional reduction in a unit time is equal to (P_t = 1_-P_0_)/P_0 _= 1-e^-K^. The parameter K is the clearance rate constant and is equal to the minus slope of the log_e _parasitaemia-time linear relationship (that is, K > 0) as log_e_(P_t_) = log_e_(P_0_) - K×time. The clearance rate constant is calculated after the tail and lag phase have been identified and removed (see Figure 2).

**Slope half-life - **the time needed for parasitaemia to be reduced by half during the log-linear phase of parasite clearance. This is a constant independent of the starting value of parasitaemia since reduction in parasitaemia follows the first order process (after excluding lag phase and tail). Half life can be calculated from the formula

$${\text{T}}_{\text{1}∕\text{2}}=\text{lo}{\text{g}}_{\text{e}}\left(\text{2}\right)∕\text{K}=0.\text{692}∕\text{K},\text{ where K is the clearance rate constant}.$$

Serial parasitaemia-time data obtained from several published studies carried out between 2001 and 2010, at sites located in Tak province in north-western Thailand, Chittagong and Bandarban in Bangladesh, Savannakhet province in Laos, western Cambodia and Mali were used in the analysis. All studies investigated the use of artemisinin combination therapies. The algorithm was also tested on data from patients treated with quinine, enrolled in a study in Ho Chi Minh City, Vietnam between 1991-1996.

When there were no zero parasitaemias recorded, normal regression was used and all recorded measurements were included in the analysis. When there were zero parasitaemias recorded tobit regression [20] was used, however only the first zero sustained (i.e. followed by negative slides only) was included in the analysis. Briefly, a tobit model is a censored regression model, where the response variable Y_i _is related to a latent (and sometimes unobservable) variable ${\text{Y}}_{\text{i}}^{*}$ by:

$${\text{Y}}_{\text{i}}={\text{Y}}_{\text{i}}{}^{*}\text{if}\;{\text{Y}}_{\text{i}}{}^{*}>{\text{Y}}_{\text{L},}{\text{Y}}_{\text{i}}={\text{Y}}_{\text{L}}\text{if}\;{\text{Y}}_{\text{i}}{}^{*}\leq {\text{Y}}_{\text{L}.}$$

The latent variable is modelled in the standard way: ${\text{Y}}_{\text{i}}^{*}={\text{X}}_{\text{i}}\beta +\varepsilon$. The regression model is solved using maximum likelihood techniques, however the likelihood function is now a truncated normal distribution in the case when ${\text{Y}}_{\text{i}}^{*}\leq {\text{Y}}_{\text{L}}$.

For each patient's parasitaemia data, the following polynomial models of time were fitted:

$$\log_{\text{e}}\left({\text{P}}_{\text{i}}\right)={\text{X}}_{\text{i}}\beta +\varepsilon_{\text{i}}$$

Where P_i _is the parasite count per microlitre measured at time t_i _and ε_i _is the corresponding error term associated with this measurement. β is a vector of unknown parameter values and X_i _is a vector of covariate values as follows:

1. Linear: X_i _= [1, t_i_], β = [b_0_, b_1_]

2. Quadratic: ${\text{X}}_{\text{i}}=\;\left[\text{1},\;{\text{t}}_{\text{i}},{\text{ t}}_{\text{i}}^{2}\right]$, β = [c_0_, c_1_, c_2_]

3. Cubic: ${\text{X}}_{\text{i}}=\;\left[\text{1},\;{\text{t}}_{\text{i}},{\text{ t}}_{\text{i}}^{2},{\text{t}}_{\text{i}}^{3}\right]$, β = [d_0_, d_1_, d_2_, d_3_]

For subjects with only four positive parasite measurements, the cubic regression model could not be fitted so a linear regression model starting from the second parasite measurement was fitted if this second measurement exceeded the first measurement (at time 0) by more than 25%. These "maximum regression" models often fitted better than the quadratic models that typically do not fit well for data with a steep increase in parasitaemia.

Linear, quadratic and cubic models fitted to the same data were compared using Akaike Information Criterion (AIC) [21] and the model that minimised the AIC was selected as best describing changes in log-parasitaemia over time. For subjects with a "maximum regression" model fitted, the sum of the squared residuals for the observations common to all three models (RSS_shared_) was used instead and the model that minimised the RSS_shared _was selected. From the selected best fitting model the lag phase was identified and the clearance rate constant was estimated using the algorithm described below.

A sensitivity analysis was also performed to assess the effect of parasite sampling times, the exclusion of lag-phase from the estimation, and the use of tobit regression on estimates of the slope half-life. In order to do this, slope half-life estimates were also calculated using the following methods:

1. as described in this paper on a subset of patients with measurements taken every six hours in the first two days; with every second data-point excluded from the estimation;

2. linear regression (tobit or normal regression as appropriate), i.e. ignoring the lag phase;

3. normal regression where all zero-counts were excluded from the estimation, but the lag-phase was evaluated as described.

Each of the results from the above models (denoted here by B) were compared with results from fitting models using the methodology described in this paper (denoted here by A). The relative difference in slope half-life was calculated as:

$$\left({\text{Slope half-life}}_{\text{B}}-{\text{Slope half-life}}_{\text{A}}\right)∕{\text{Slope half-life}}_{\text{A}}\times 100\%$$

### Algorithm

Before model selection and fitting, the data are checked for problem data points, possible outliers, and persistent parasite tails in an automated fashion. The data cleaning process is outlined briefly in Additional File 1. Having determined that it is appropriate to fit a model to the subject's parasite count data, the model fitting and estimation of the clearance rate constant (K) and duration of lag phase (t_lag _) is performed. The methodology is summarized in the following steps:

For each patient separately:

**Step 1: **Perform data cleaning, as described in Additional File 1. All further steps are performed on data with outliers, tails, extreme values and trailing zeros removed. For convenience of notation, if there is a zero parasitaemia that directly follows the last positive parasitaemia, it is replaced with the detection limit. This data point is subsequently counted as a non-zero parasite count.

**Step 2: **Perform checks to see if the clearance rate constant cannot be estimated:

(i) Number of non-zero parasite measurements (including a zero replaced with the detection limit) less than three

(ii) Initial parasitaemia too low: initial parasitaemia < 1,000 parasites per microlitre

(iii) Final recorded parasitaemia too high: final parasitaemia ≥ 1,000 parasites per microlitre

(iv) A zero parasitaemia has been recorded, but the last positive parasitaemia is too high and the zero count is uninformative: last non-zero parasitaemia ≥ 1,000 parasites per microlitre

A zero parasitaemia is defined to be uninformative if the normal linear regression fitted to all the data points (excluding the zero count) gives a confidence interval for the time when the parasitaemia is below the level of detection which includes the time-point when the zero parasitemia was recorded.

**Step 3**: Perform additional check to see if there are not enough data to estimate a lag phase

(v) There are fewer than three measurements in the first 24 hours or a time difference between measurements in the first 24 hours is more than 14 hours;

**Step 4: **Perform model fitting.

**Figure 3:**
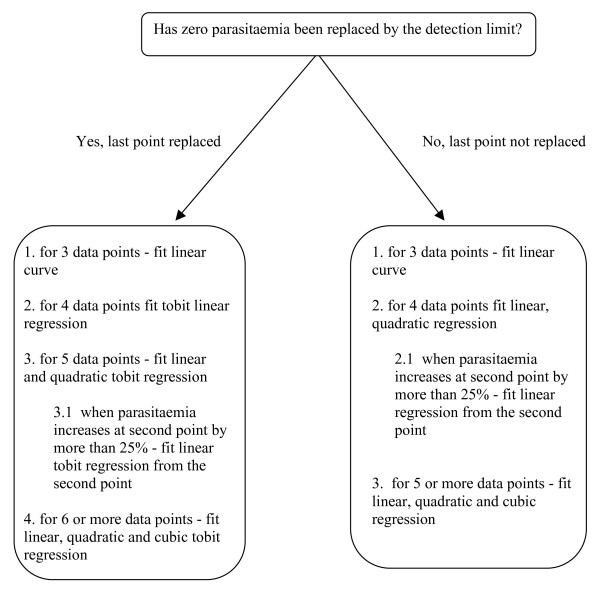
**Flow chart showing the models tested during the process of finding the best model representing parasite clearance over time for an individual patient**.

If none of the exclusion criteria (i)-(iv) in **Step 2 **and (v) in **Step 3 **are satisfied, fit polynomial models to the natural log-parasitaemia *versus *time data as described in Figure 3.

If none of the exclusion criteria (i)-(iv) in **Step 2 **are satisfied but exclusion criterion (v) in **Step 3 **is satisfied fit tobit linear regression if a zero parasitaemia has been recorded or linear regression otherwise.

If one or more of the exclusion criteria are satisfied in **Step 2**, the clearance rate constant and duration of lag phase cannot be estimated so proceed to **Step 6**.

**Figure 4:**
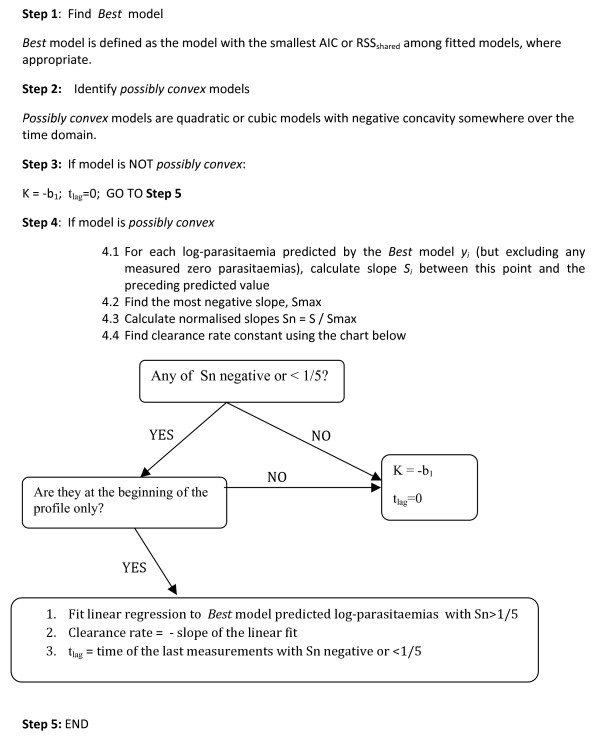
**Diagrammatic representation of the process of estimating the clearance rate constant (K) and the duration of lag phase (t_lag _) from individual patient parasite counts measured over time**.

**Step 5: **Estimate clearance rate constant (K) and duration of lag phase (t_lag _) as described in Figure 4.

**Step 6: **END

### Estimation of clearance rate constant and duration of lag phase

The procedure to estimate the parasite clearance rate constant depends on the shape of the parasite profile, i.e. the shape of the *best *polynomial model fitted and the amount of data available. For profiles with only three parasite counts, infrequent parasite measurements in the first 24 hours, profiles which are linear or exhibit concave curvature, the minus slope of the linear regression model or tobit linear model (where appropriate) is used as an estimate of the clearance rate constant. Profiles which exhibit convex curvature are examined further to define the lag phase. If a lag phase is not identified in the profile, then the minus slope of the linear regression model or tobit linear model is used as an estimate of the clearance rate constant.

For profiles with convex curvature the assessment is performed on the *best *model predicted values with respect to changes in *pair-slopes *- slopes calculated between two neighbouring data points. Most *pair-slopes *are negative since the parasitaemia is decreasing and the smallest slope corresponds to the fastest parasite clearance. First, the minimum possible *pair-slope *is found for the entire profile within the time interval of detectable parasite measurements. If the profile is linear then all *pair-slopes *would be similar to this minimum slope, but if there is pronounced curvature in the profile, then there would be *pair-slopes *which are near to zero (corresponding to the flat part of the curve). If this flat part of the curve occurs at the initial time-points then it is called the lag phase and is excluded from the estimation of the parasite clearance rate constant. However the data-point which is the upper limit for the lag phase period is included in calculations (i.e. if the duration of lag phase is 12 hours then the data-point at 12 hours is included in the final estimation of the clearance rate constant).

## Results

The algorithm was tested on a large data set, consisting of 4,236 individual patient parasite-time profiles from clinical studies of ACT: artesunate monotherapy, artesunate and mefloquine, artemether and lumefantrine, artesunate and amodiaquine. The median number (range) of positive parasite slide readings was seven (three-33) per patient. The detection limit was 16 (in 81% of patients), 25 (in 4% of patients) or 40 (in 15% of patients) parasites per microlitre. Within this large data-set, 4,008 profiles had their parasite clearance rates calculated, of which 294 profiles were best fitted with a quadratic model and a non-zero lag phase, 883 profiles were best fitted with a cubic model (and non-zero lag phase), and 2,821 were best fitted with a linear model (either directly (1,146 profiles), or by a quadratic model with zero lag phase (704 profiles) or by a cubic model with zero lag phase (971 profiles)). The breakdown of fitted models is provided in Table 1. We note that of the 2,831 fitted with a linear model, 55 of these had only 3 time points (in which case the only option was to fit a linear model).

**Table 1:** Breakdown of models fitted.

	Number of models fitted (% of total)
Linear fit	1146 (28.6%)

Max reg fit	10 (0.3%)

Quadratic fit (total)	998 (24.9%)
t_lag _= 0	704 (17.6%)
t_lag _> 0	294 (7.3%)

Cubic fit (total)	1854 (46.2%)
t_lag _= 0	971 (24.2%)
t_lag _> 0	883 (22.0%)

Of the 228 profiles that did not pass the requirements for clearance rate estimation, 112 were not fitted because of a lack of data (< 3 positive parasite readings), 27 because the initial parasitaemia was too low, and 89 because the parasitaemia was not cleared (last recorded parasitaemia was above 1,000 parasites per microlitre). Among 4,008 patients for whom estimation could be performed, tails were identified in 1,070 profiles (27%). In the automated cleaning process, a total of 31 outliers were identified. These points were not included in any subsequent clearance rate calculations. A lag phase was estimated in 30% (1,187/4,008) of profiles; the median (IQR; range) duration of lag phase was six (6-9; 1-60) hours.

### Summary of model outputs and examples of fit

For the 4,008 profiles fitted, the median (IQR; range) parasite clearance rate constant was 0.22 (0.16-0.30; 0.02-1.18) per hour. The corresponding slope half-life had a median (IQR; range) of 3.11 (2.33-4.24; 0.59-34.28) hours. It should be noted that the data used in this paper has come from multiple study sites, across a range of patient demographics and, as such, should not be used as a reference point for comparison to other parasite clearance data.

**Figure 5:**
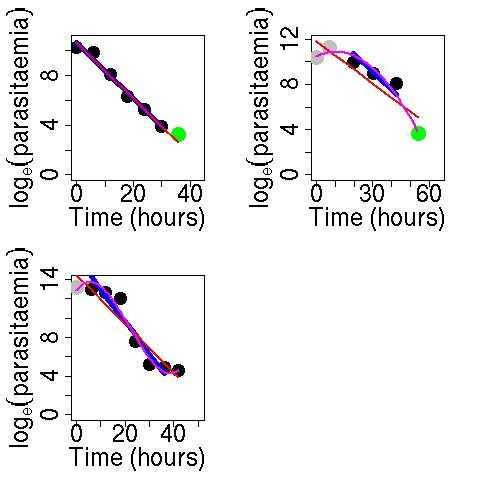
**Linear and polynomial fits to the quadratic or cubic -shaped log(parasitaemia)-time profiles shown in Figure 3**. Black points represent the data, green points represent censored values and grey points represent points identified as being part of the lag phase. Pink line shows cubic fit to the data, red line shows linear fit the data and blue line shows the linear fit to the identified 'linear part' of the profile.

Figure 5 shows examples of individual log-parasitaemia - time profiles, with linear and polynomial fits to the data. The clearance rate constants calculated by a linear fit applied to all data points were consistently different to the clearance rate constants calculated by accounting for the lag phase (detailed comparisons between the slope half-life calculated using the lag regression technique to those calculated by fitting a linear model to the data are presented below).

### Goodness of fit

**Figure 6:**
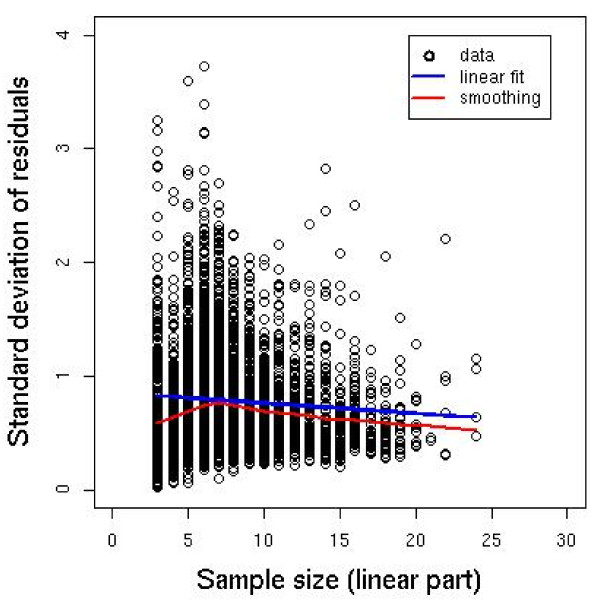
**The standard deviation of the residuals *versus *the sample size of the linear part of the parasite clearance profile**.

Agreement between predicted and measured log-parasitaemia was better for the final linear models derived from the polynomial models than for the linear regression models fitted to all data points: the residuals had smaller variation over the data used to calculate the clearance rate constants: their mean (standard deviation) for the time points included in estimation of the clearance rate constant was 0.00 (0.42) for the polynomial models and 0.09 (0.80) for the corresponding linear models. Figure 6 shows a plot of the standard deviation of the residuals for all profiles fitted *versus *the number of data points used to calculate the slopes of the linear part of the parasite clearance profiles. Overall, as expected, there is a decrease in the variability of standard deviation of the residuals as the number of parasite counts in the linear part of the parasite clearance profile increases, although the median value of the standard deviation of the residuals remains the same as sample size increases.

### Comparison between lag regression and linear regression

**Figure 7:**
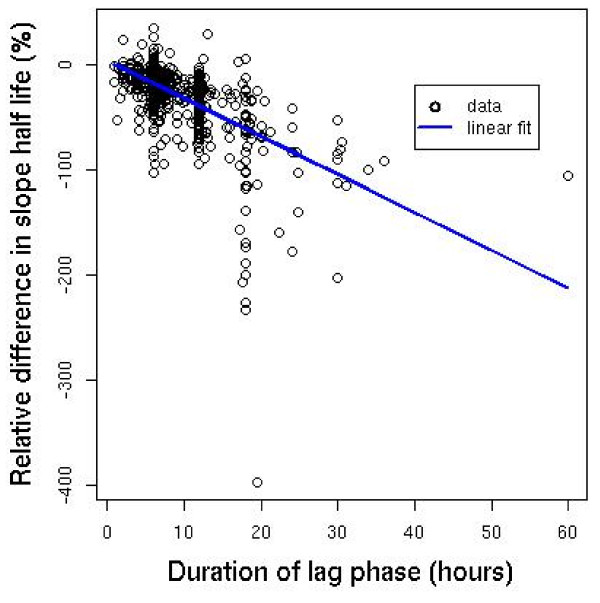
**The relative difference in slope half-life rate comparing the final slope half-life to that calculated by fitting a linear model to all the data**. (Note that only those profiles for which t_lag _> 0 were used here, for comparison purposes). Relative difference in slope half-life is calculated as the slope half-life from lag regression minus the slope half-life from linear regression, divided by the slope half-life from lag regression.

Among profiles with a lag phase (30%; n = 1187) identified, the linear regression models tended to overestimate the slope half-life; the median (IQR; range) of the relative difference in slope half-life was -18% (-28% to -11%; -397% to 35%). The differences decreased with the increased duration of the lag phase (Figure 7). For the subset of data where a lag phase of approximately six hours duration was estimated, the median (IQR; range) relative difference in the slope half-life was -15% (-22 to -10%; -102% to 35%) as compared to -76% (-114% to -52%; -397% to 6%) for a lag phase of approximately 18 hours or more.

### Frequency of sampling

**Figure 8:**
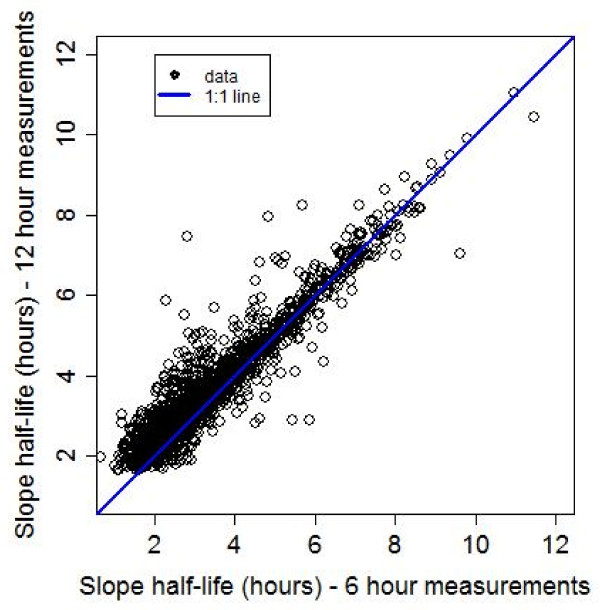
**The comparison between slope half-life estimated from parasite measurements taken every six and 12 hours in the first two days**.

**Figure 9:**
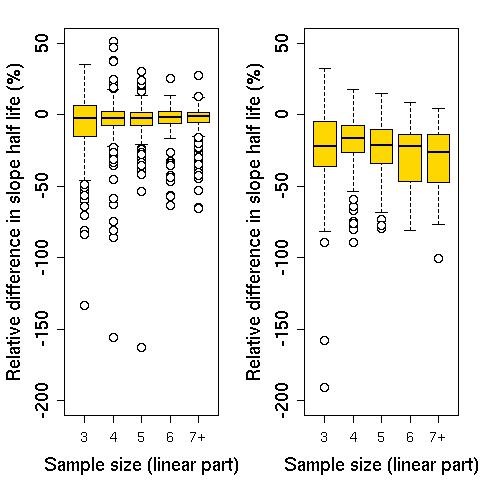
**Relative difference in slope half-life estimated from six-hourly and 12-hourly measurements, by sample size**. Left panel shows results for profiles with no lag phase identified based on six-hourly measurements; right panel shows results for profiles with lag phase. Sample size is equal to number of measurements at 12-hourly intervals in linear part of profile (after excluding lag phase and tails). Relative difference in slope half-life is calculated as the slope half-life from six-hourly measurements minus the slope half-life from 12-hourly measurements, divided by the slope half-life from six-hourly measurements.

**Figure 10:**
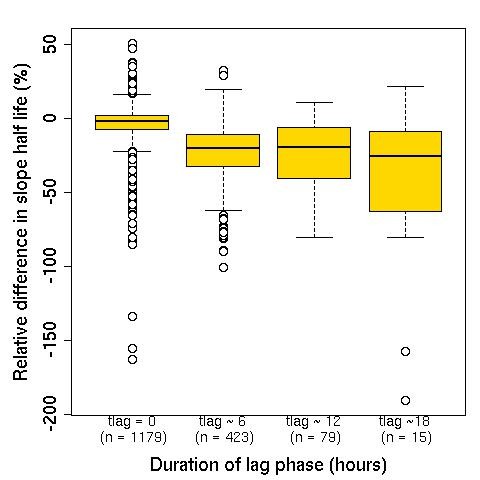
**Box-plots of relative difference in slope half-life estimated from six-hourly and 12-hourly measurements, stratified by lag phase (t_lag_)**. Relative difference in slope half-life is calculated as the slope half-life from six-hourly measurements minus the slope half-life from 12-hourly measurements, divided by the slope half-life from six-hourly measurements.

Among all patients for whom estimation was performed, 1,715 had parasite counts taken at regular six-hour intervals in the first 48 hours. In order to assess whether less frequent sampling would be adequate for slope estimations, the clearance rate constant and slope half-life were estimated after removal of every second data point (i.e. including only parasite counts taken every 12 hours) and compared with the estimates based on all data (Figure 8). The agreement between estimates was better for profiles without a lag phase and with a minimum of four counts in the linear segment of the profile (after excluding lag phase and tail) (Figures 9 and 10). Slope half-life tended to be overestimated when 12 hourly measurements were used. The median (IQR; range) relative difference in slope half-life was -4.8% (-17.3% to 1.2%; -190.9% to 50.9%). The difference in slope half-life was mostly due to (a) unidentified lag phase when 12 hourly measurements were used and (b) delayed record of zero parasite count. In both situations a line with a flatter slope was fitted compared to the fit with six hourly data.

For profiles with no lag phase identified by six-hourly measurements, the percentage of profiles with an absolute relative difference in slope half-life above 10% was 19% for sample size of four or more counts (of the linear part of the 12 hour data), decreasing to 7% for a sample size of at least six and 0.6% for nine or more data points. The corresponding figures for an absolute difference of 20% or more were 7%, 3% and 0.3%.

If a lag phase was identified using six-hourly measurements, the relative differences in slope half-life was greater; 21% of profiles with a sample size of four or more in the linear segment of the 12-hour data were different by more than 30%. The estimated duration of lag phase did not change in 1,204 of the 1,696 profiles (71%). However, of these 1,204 profiles 1,167 (97%) were profiles with no lag phase identified by both models (Table 2).

**Table 2:** Summary of changes in lag phase when 12-hourly parasite counts were used instead of six-hourly counts.

	**Duration of lag phase (hours)**^**a**^			
**Change in t**_**lag **_	**0 hours**^**b**^	6 hours	12 hours	**18 hours**^**c**^
Decreased	0 (0%)	354 (84%)	42 (53%)	12 (80%)

Remains unchanged	1167 (99%)	0 (0%)	37 (47%)	0 (0%)

Increased	12 (1%)	69 (16%)	0 (0%)	3 (20%)

Values presented in brackets is the percentage of the column sum.

^a^number of patients (%) given

^b^no lag phase was identified

^c^duration of lag phase of 18 hours or longer

Of 517 profiles with a lag phase detected by the six-hourly measurements, the majority (79%) had the estimate of the duration of lag phase shortened when 12-hourly measurements were used, resulting in a systematic overestimation of the slope half-life. The median (IQR; range) relative difference in slope half-life for those profiles that had both a lag phase detected with the six-hourly measurements and this lag phase shortened under the 12-hourly measurements was -25.1% (-40.2% to -16.0%; -190.9% to 28.8%). Overall this analysis indicates that parasite clearance profiles with a lag phase are poorly characterised if the first parasite count after baseline is taken at 12 hours.

### Use of tobit regression

The algorithm was tested on the 2,401 profiles which required tobit regression. The median relative difference in slope half-life between tobit regression and normal regression was -0.01% with a range of (-134% to 71%). Overall 14.4% of slopes had an absolute difference of more than 10% and only 4.4% of profiles had a difference of more than 30% (Table 3). Even though there is little overall difference between estimates when normal regression was used instead of the tobit regression, in cases when the last recorded parasitaemia is high the difference could be substantial.

**Table 3:** Agreement between tobit regression and linear regression estimates of slope half-life.

	**Relative Difference in Slope half-life**^**a**^		
**Sample size**^**b**^	< 10%	< 30%	< 50%
3 < N ≤ 5	620 (90%) (26, 44)	669 (97%) (9, 12)	683 (99%) (5, 2)

5 < N ≤ 10	1104 (81%) (19, 242)	1281 (94%) (2, 82)	1352 (99%) (1, 12)

10 < N ≤ 15	331 (96%) (7, 8)	346 (100%) (0, 0)	346 (100%) (0, 0)

The two numbers in brackets represent the percentage within the threshold and the number of cases below and above the margin respectively.

^a ^Number of patients (%) within margin and (number of patients below margin, number of patients above margin) are given

^b ^Number of observations in the linear part of the parasite clearance profile

### Different anti-malarial drug treatments

The algorithm was tested on data from a study of 382 patients investigating the effect of quinine on parasite clearance. The median number (range) of positive parasite counts from slide readings was seven (three-30) per patient. Overall, 285 profiles were suitable for estimation of parasite clearance rates, of which 36 profiles were best fit with a quadratic model and a nonzero lag phase, 70 profiles were best fit with a cubic model and nonzero lag phase, and 179 were best fit with a linear model (either directly, or by a higher order model with zero lag phase). Of the 97 profiles that did not pass the requirements for clearance rate estimation, four were not fitted because of lack of data (< 3 positive parasite counts), 15 because the initial parasitaemia was too low, and 78 because the parasitaemia was not cleared (last recorded parasitaemia was above 1,000 parasites per microlitre). Among the patients for whom estimation could be performed, tails were identified in 45 profiles (16%) and 106 had a non-zero lag phase (37%).

Overall, the median (IQR; range) slope half-life for patients treated with quinine was 5.15 (3.83-6.68; 0.86-105.5) hours and, as expected, was longer than for ACT. Median (IQR; range) slope half-life for ACT was 3.11 (2.33-4.24; 0.59-34.28) hours. Patients treated with quinine were more likely to have a lag phase (37% *versus *30% in the ACT group) and the duration of lag phase was significantly longer: median (IQR; range) of 10.0 (6.31-18.13; 0.5-81.97) hours as compared to six (6-9; 1-60) hours in ACT group.

For the quinine data-set, as with the data-set from patients treated with ACT, the agreement between the predicted and measured log-parasitaemia was better for the final linear models derived from the polynomial models than for the linear regression models fitted to all data points: the residuals had smaller variation over the data used to calculate the clearance rate constant: the mean (standard deviation) for the time points included in estimation of the clearance rate constant was 0.00 (0.39) for the polynomial models and 0.17 (1.08) for the corresponding linear models.

## Conclusions

Prolonged parasite clearance is the only way artemisinin resistance can be identified currently. In this paper a new standardized approach to the estimation of the malaria parasite clearance rate has been presented. The algorithm takes into consideration the potentially confounding effects of the lag phase and removes these initial data before calculating the rate constant of the log-linear phase of parasite clearance. An outline of the algorithm is presented and its use has been evaluated with a large series of serial parasite counts from treatment studies in patients with acute falciparum malaria. Current evidence suggests that the main effect of artemisinin resistance is on the slope of the log-linear decline and for this reason this paper has focussed on characterizing this variable. The slope measure which may be most readily interpreted by malariologists is the half-life (which is inversely proportional to the first order rate constant).

The clinical phenotype of delayed parasite clearance is likely to remain the main proxy of artemisinin resistance until molecular markers are identified or *in vitro *methods adapted which provide better characterization. Parasite clearance rates are affected by drug blood concentration profiles as well as their pharmaco-dynamic properties. Immunity also increases parasite clearance rates. Other factors such as splenic function, age, anaemia and co-infections may also affect parasite clearance and require further investigations.

Whilst artemisinin resistance in *P. falciparum *is confined to a relatively small geographic area in SE Asia, it is important to establish in other areas baseline parasite clearance data for the different ACT provided through the public sector, so trends can be followed prospectively across malaria endemic countries. The accurate assessments of parasite clearance in this "early warning system" require frequent blood sampling and accurate counting and the additional workload and the necessary quality assurance of such monitoring measures will undoubtedly raise serious operational challenges.

How frequent do parasite counts need to be to provide reliable calculation of estimates is still unclear. Measuring parasitaemia every 12 hours after starting treatment until counts are below the level of detection, allows the calculation of parasite clearance rates but systematically overestimates the slope half-life compared to measurement every six hours. The difference observed is greater when there is a lag phase, and is more evident with certain anti-malarials, e.g. quinine compared to artemisinin derivatives.

More data are needed to estimate the optimal sampling design for parasite clearance characterisation. However, considering the field challenges of performing systematic measures of parasitaemia more frequently than 12-hourly, this sampling interval could be considered as the minimum.

The WorldWide Anti-malarial Resistance Network (WWARN) has developed the Parasite Clearance Estimator or PCE (see http://www.wwarn.org/research/parasite-clearance-estimator for details), which uses the methodology described above to estimate parasite clearance measures. WWARN aims at providing a platform of partnership, which facilitates gathering the necessary data across endemic countries, needed to answer to the different methodological questions raised above.

Identifying the clearance rate constant and lag phase with the improved method outlined here will provide necessary standardized information in order for warning signs of resistance to be detected, so that appropriate actions to contain resistance can be initiated [6].

## Competing interests

The authors declare that they have no competing interests.

## Authors' contributions

KS, NW and PG designed the study. JF and KS implemented the algorithms. JF and KS drafted the manuscript. NW and PG edited the manuscript. All authors read and approved the final manuscript.

## Supplementary Material

Additional file 1 **Data cleaning process for the parasite clearance estimator**. The cleaning process implemented in the algorithm is described, including removal of recurrence parasitaemia, trailing zeros, tails, replacement with the detection limit, extreme values and outliers. Click here for file
