# Supplementary material for: Standardizing the measurement of parasite clearance in falciparum malaria: the parasite clearance estimator
Source: Malar J. 2011 Nov 10;10:339. doi: 10.1186/1475-2875-10-339 (PMC3305913; doi:10.1186/1475-2875-10-339)
Supplement: Additional file 1 — Data cleaning process for the parasite clearance estimator. The cleaning process implemented in the algorithm is described, including removal of recurrence parasitaemia, trailing zeros, tails, replacement with the detection limit, extreme values and outliers. [file 1475-2875-10-339-S1.DOCX]

Data cleaning

The entire process of data cleaning is performed in the following order:

1. Removal of data from recurrences of parasitaemia

Any measurements after 7 days, data after a time when two consecutive measurements are longer than 24 hours apart and data after the last measured zero parasitaemia if the positive measurements proceeding and preceding it are separated by more than 24 hours are removed.

1. Removal of trailing zeros

All zero values after the last non-zero parasitaemia, accepting the very first zero are removed.

1. Removal of tails

Repeated parasitaemias below 100/uL and data that fall between them are removed.

1. Replacement of the first 0 value with the detection limit (DT)

If there is a zero parasitaemia that directly follows the last positive parasitaemia, the zero is replaced with the detection limit.

1. Removal of extreme values

Times below 0 and/or values of parasitaemia outside of the range (0, 3 x 10^6^) are removed.

1. Removal of outliers

Parasite measurements which are not consistent with the two immediately preceding and succeeding measurements are removed. “Consistent" is defined by the comparison of the rate of change in parasitaemia to the average rate in the profile.

The normalized slope between each set of neighbouring points is calculated by taking the ratio of the rate of change between each set of two consecutive points to the average rate of change over the entire profile.

In the first 12 hours a data point that is associated with the following conditions is removed

*NormSlope _i_*  < - 20 and *NormSlope _i +1_* >10

where *NormSlope_i_* denotes the normalized slope between the *i^th^* and (*i*+1)^th^ data points.

After the first 12 hours this is relaxed so that now points associated with either of the following two conditions are removed.

*NormSlope _i_*  < - 7.5 and *NormSlope _i +1_* >10

or

*NormSlope _i_*  < - 40 and *NormSlope _i +1_* >3.75

Furthermore, at any stage during the parasite clearance points that are associated with any of the following 4 conditions are removed.

(i) *NormSlope _i_*  >2 and *NormSlope _i +1_* < -10

(ii) *NormSlope _i_*  >10 and *NormSlope _i +1_* < -2

(iii) *NormSlope _i_*  >1 and *NormSlope _i +1_* < -20

(iv) *NormSlope _i_*  >50 and *NormSlope _i +1_* < 0.4

Finally, the last parasitaemia is considered to be an outlier if the second last parasitaemia is less than 200 and the last is more than three times the second last and more than 100.

Initially these thresholds were defined based on an example data set (with approximately 100 profiles) with “noisy” parasite measurements extracted from the data of more than 3000 serial parasitaemia–time profiles. For this subset of data the 4 authors decided independently on what would classify as an outlier and the values of the thresholds were adjusted to meet these decisions.
